# Decoding Diagnostic Delay in COPD: An Integrative Analysis of Missed Opportunities, Clinical Risk Profiles, and Targeted Detection Strategies in Primary Care

**DOI:** 10.3390/diagnostics15172209

**Published:** 2025-08-30

**Authors:** Juan Luis Rodríguez Hermosa, Soha Esmaili, Iman Esmaili, Myriam Calle Rubio, Carla Novoa García

**Affiliations:** 1 Pulmonology Department, Hospital Clínico San Carlos, 28003 Madrid, Spain; jlrhermosa@yahoo.es (J.L.R.H.); carlagn7@gmail.com (C.N.G.); 2 Instituto de Investigación Sanitaria del Hospital Clínico San Carlos (IdISSC), 28003 Madrid, Spain; soha@esmaili.ws; 3 Department of Medicine, School of Medicine, Universidad Complutense de Madrid, 28040 Madrid, Spain; 4 School of Medicine, Universidad Antonio de Nebrija, 28240 Madrid, Spain; 5 Heart Lung Innovation Centre, Vancouver, BC V6Z 1Y6, Canada; 6 International Science Novelty Services (ISNS) Data Analytics and Research, Vancouver, BC V6B 1J6, Canada; iman@esmaili.ca; 7 CIBER de Enfermedades Respiratorias (CIBERES), 28029 Madrid, Spain

**Keywords:** COPD, diagnostic delay, missed opportunities, primary care, predictive modeling, cluster analysis, early detection

## Abstract

**Background**: Delayed diagnosis of Chronic Obstructive Pulmonary Disease (COPD) in primary care is common and contributes to preventable morbidity. A deeper understanding of pre-diagnostic patterns is needed to develop targeted detection strategies. We aimed to characterize diagnostic delay and missed diagnostic opportunities (MDOs) and identify high-risk clinical profiles. **Methods**: We conducted a retrospective cohort study of 167 patients newly diagnosed with COPD in primary care centers in Madrid, Spain. Healthcare utilization in the 12 months preceding diagnosis was analyzed. Multivariable logistic regression was used to identify predictors of MDOs, and K-means clustering was used to identify patient phenotypes. **Results**: Diagnostic delay (>30 days) was present in 45.5% of patients, and MDOs in 47.3%. MDO-positive patients had significantly worse lung function (mean FEV_1_: 1577 vs. 1898 mL, *p* = 0.008), greater symptom burden (CAT score ≥ 10: 79.7% vs. 59.1%, *p* = 0.003), and more frequent pre-diagnostic exacerbations (mean: 1.24 vs. 0.71, *p* = 0.032). After multivariable adjustment, diagnostic delay remained a powerful independent predictor of MDOs (OR 10.25, 95% CI 4.39–24.88; *p* < 0.001). Cluster analysis identified three distinct clinical phenotypes: ‘Paucisymptomatic–Preserved’, ‘Frequent Attenders/High-Risk’, and ‘Silent Decliners’. **Conclusions**: The pre-diagnostic period in COPD is a dynamic window of detectable, and potentially preventable, clinical deterioration driven by diagnostic inertia. The identification of distinct patient phenotypes suggests that a proactive, stratified, and personalized approach, rather than a one-size-fits-all strategy, is required to improve early diagnosis in primary care.

## 1. Introduction

Chronic Obstructive Pulmonary Disease (COPD) is a leading cause of morbidity and mortality worldwide, characterized by persistent respiratory symptoms and airflow limitation due to airway and/or alveolar abnormalities, often caused by significant exposure to noxious particles or gases [1]. Despite substantial advancements in diagnostic criteria and management guidelines, the timely detection of COPD remains a major clinical challenge, particularly in Primary Care (PC) settings where most patients initiate their diagnostic journeys. Early detection and intervention are essential to improve clinical outcomes, reduce disease progression, enhance quality of life, and optimize healthcare resource utilization [2]. Delayed diagnosis, however, continues to be pervasive, contributing to poor clinical outcomes, increased healthcare costs, and reduced effectiveness of therapeutic interventions [3].

PC plays a critical role in the early detection of COPD, as it is the first point of contact for most symptomatic patients [4]. Studies have shown that PC providers are often the initial healthcare professionals consulted by individuals with early symptoms of COPD, including dyspnea, chronic cough, and sputum production [5]. However, several factors contribute to the frequent under-recognition of COPD in these settings, including limited access to spirometry, lack of adherence to diagnostic guidelines, and insufficient clinical awareness of the disease [6]. The failure to recognize and diagnose COPD at earlier stages often results in missed opportunities for intervention, particularly in the context of PC, where diagnostic processes typically originate [7].

The implications of delayed diagnosis in COPD are profound [8]. A significant proportion of patients remain undiagnosed until advanced stages of the disease [9], thereby limiting the potential benefits of early intervention strategies such as smoking cessation [10], pharmacological treatment, and pulmonary rehabilitation [11]. A delayed diagnosis has been associated with greater symptom burden, reduced lung function, and an increased risk of exacerbations and hospitalizations, all of which contribute to poorer overall prognosis [12]. Moreover, missed opportunities for diagnosis in PC are especially problematic, as they represent critical points where early detection could have been achieved but was not [13]. Understanding and addressing these missed opportunities is essential for improving COPD outcomes, particularly given the rising burden of the disease worldwide.

Previous studies have highlighted the negative impact of diagnostic delays and have developed predictive models to identify patients at risk [14]. However, a deeper understanding of the complex patient journeys preceding a formal diagnosis is still needed [15].

Therefore, this study was designed to characterize the clinical profiles and healthcare utilization patterns associated with different diagnostic timelines in a real-world cohort of patients with newly diagnosed COPD. The specific objectives were (1) to describe the clinical, functional, and healthcare utilization characteristics of patients according to their pre-diagnostic pathway; (2) to quantify the association between these pre-diagnostic patterns and the clinical burden at the time of diagnosis; and (3) to use exploratory data-driven methods to identify distinct patient profiles, recognizing that any observed associations are likely influenced by underlying disease severity and other potential confounders. This approach aims to provide a detailed descriptive analysis to better inform the design of future prospective studies on early COPD detection.

## 2. Materials and Methods

### 2.1. Study Design and Setting

This retrospective cohort study was conducted in Primary Care (PC) centers in Madrid, Spain, to evaluate patterns of healthcare utilization, diagnostic delay, and missed diagnostic opportunities (MDOs) during the 12 months preceding a new diagnosis of COPD. The inclusion period for newly diagnosed patients spanned from September 2022 to September 2023. A formal diagnosis of COPD was established according to the Global Initiative for Chronic Obstructive Lung Disease (GOLD) criteria, defined as a post-bronchodilator Forced Expiratory Volume in 1 s (FEV_1_) to Forced Vital Capacity (FVC) ratio of <0.70 [16]. The retrospective design was selected to robustly characterize the temporal sequence of clinical events and healthcare interactions leading to diagnosis.

### 2.2. Study Population

The study population included patients aged ≥40 years with a new, spirometry-confirmed diagnosis of COPD, a smoking history of ≥10 pack-years, and at least 12 months of comprehensive pre-diagnostic data available in their electronic medical records. Patients were excluded if they had a pre-existing diagnosis of other chronic respiratory conditions that could confound the analysis, such as asthma, bronchiectasis, or interstitial lung disease, or if their pre-diagnostic healthcare data were incomplete. The patient selection and exclusion process is detailed in the study flow diagram (Supplementary Figure S1).

### 2.3. Data Collection and Variable Definitions

The information collected was concurrent in nature for clinical data obtained during the single visit conducted at the time of inclusion and diagnosis, and historical for the assessment of health interactions prior to COPD diagnosis, extracted from a systematic review of structured electronic medical records (EMRs) and subsequently verified by clinical researchers. The collected variables included demographic characteristics and clinical data at the time of diagnosis, such as smoking status and Body Mass Index.

During this structured inclusion visit, patients provided written informed consent and completed validated clinical questionnaires under supervision. Specifically, the CAT, mMRC, and IPAQ-SF scores were filled out by patients with the assistance of trained research staff, and the AVD score was obtained through a structured clinical interview. These instruments were pre-specified in the study protocol and administered as part of the standardized assessment at the time of diagnosis.

Symptom burden was assessed using the COPD Assessment Test (CAT), a validated 8-item questionnaire widely recommended to quantify disease impact [17]. Dyspnea severity was measured with the modified Medical Research Council (mMRC) scale, a standard tool for functional classification in COPD [18]. Physical activity was evaluated using the short-form International Physical Activity Questionnaire (IPAQ-SF), a reliable and validated instrument in COPD populations [19]. Clinical phenotyping and risk stratification were defined according to the Spanish COPD Guidelines (GesEPOC 2021) [20]. Functional limitation in daily life was captured through an adapted Activities of Daily Living (AVD) score based on prior Spanish COPD cohorts. Although not formally validated, this pragmatic index has previously demonstrated meaningful associations with symptom severity and autonomy [21]. The frequency of pre-diagnostic exacerbations, defined as any acute respiratory event requiring systemic corticosteroids or antibiotics or prompting an unscheduled medical visit, was also recorded.

Key analytical variables were operationally defined to ensure standardized analysis. Diagnostic Delay was defined as an interval of >30 days between the first documented healthcare contact for respiratory symptoms and the formal, spirometry-confirmed diagnosis. Given the absence of a universally accepted definition for diagnostic delay in COPD, this pragmatic threshold was established to represent a clinically significant period that exceeds a typical follow-up consultation, thus serving as a marker of diagnostic inertia. This threshold was further validated by a sensitivity analysis (Appendix A, Table A3), which confirmed that the >30-day cutoff demonstrated the strongest statistical association with missed diagnostic opportunities compared to alternative thresholds (60, 90, and 120 days).

Missed diagnostic opportunities (MDOs) were quantified using a continuous Weighted MDO Score, which aggregated points from six pre-diagnostic healthcare indicators. The weighting system was designed to reflect the clinical severity of the events, assigning higher scores to those indicating greater clinical instability (e.g., hospital admission for a respiratory cause [3 points]; emergency visit or initiation of maintenance inhaled therapy [2 points each]; and a course of systemic corticosteroids, a course of antibiotics, or an unscheduled PC visit for respiratory symptoms [1 point each]), an approach conceptually analogous to clinical risk stratification tools. For stratified analyses, patients were dichotomized into MDO-positive (score ≥ 4) or MDO-negative (<4).

Finally, a composite severity score was developed to provide an integrated measure of baseline clinical burden by summing four indicators: CAT score, AVD score, mMRC grade, and a measure of airflow limitation (calculated as 100—post-bronchodilator FEV_1_% predicted). To avoid circular inference in statistical models, composite scores were not tested against their individual constituent variables.

### 2.4. Sample Size and Statistical Power

An a priori sample size calculation indicated that approximately 150 patients would be required to detect a clinically meaningful odds ratio (OR = 1.5) for the primary dichotomous outcomes with 80% power at a two-tailed α-level of 0.05. The final cohort of 167 patients exceeded this requirement, ensuring adequate statistical power for all planned stratified and multivariable analyses. The robustness of the multivariable models was further supported by adhering to the criterion of at least 10 events per predictor variable.

### 2.5. Statistical Analysis

Data were analyzed using R (version 4.1) and SPSS (version 28). All statistical tests were two-tailed, with significance set at *p* < 0.05. Descriptive statistics were used to summarize baseline characteristics. Continuous variables were reported as mean (standard deviation) or median (interquartile range) and compared using independent-sample *t*-tests or Mann–Whitney U tests, as appropriate. Categorical variables were reported as counts (percentages) and compared using the Chi-square or Fisher’s exact test.

To identify independent predictors of missed diagnostic opportunities (MDO-positive status), a multivariable logistic regression model was constructed. Covariates were selected based on clinical relevance, and model performance was assessed for discrimination and calibration, with internal validity confirmed using 10-fold cross-validation. For risk stratification purposes, a separate predictive model for diagnostic delay was developed, and patients were categorized into tertiles (low, moderate, and high risk) based on their predicted probabilities.

To identify novel patient phenotypes, an exploratory K-means cluster analysis was performed. To avoid inferential circularity, between-cluster differences were quantified using standardized effect sizes (Cohen’s d for continuous variables and Cohen’s h for categorical proportions) rather than *p*-values. The relationship between the duration of diagnostic delay and clinical severity was visualized by plotting mean indicator values across binned intervals of delay, and multidimensional clinical profiles were visualized using radar charts. To control the family-wise error rate from multiple comparisons, the Holm–Bonferroni correction was applied where appropriate. Detailed specifications of all models are provided in the Appendix A.

### 2.6. Ethical Considerations

The study protocol was approved by the Clinical Research Ethics Committee of Hospital Clínico San Carlos (code: C.I. 19/335-E; 31 July 2019). Written informed consent was obtained from all participants. The study adhered to the Declaration of Helsinki and Good Clinical Practice standards. All data were anonymized before analysis and handled confidentially.

## 3. Results

### 3.1. Baseline Characteristics by Diagnostic Pathway

The final cohort comprised 167 patients with newly diagnosed COPD. The baseline clinical, functional, and healthcare utilization characteristics of the study population, stratified by the presence of diagnostic delay and missed diagnostic opportunities, are presented in Table 1 and Table 2, respectively.

As detailed in Table 1, patients with a diagnostic delay (>30 days) presented with a significantly greater clinical burden at the time of diagnosis. This was characterized by worse airflow limitation (FEV_1_) and greater symptom burden (CAT score). While there was a trend toward a different distribution in GOLD stages between the groups, this difference did not reach statistical significance. Furthermore, this group had a significantly higher frequency of pre-diagnostic exacerbations and greater healthcare utilization across all metrics, including unscheduled primary care visits, emergency room consultations, and hospital admissions.

Similarly, patients classified as having missed diagnostic opportunities (MDO-positive) also exhibited a more severe clinical profile at diagnosis (Table 2). Compared to the MDO-negative group, they had significantly worse lung function, higher symptom scores, and were more likely to be classified as high-risk by GesEPOC criteria. A markedly higher use of pre-diagnostic respiratory medications (LABA, LAMA, and ICS) was also observed in the MDO-positive group.

### 3.2. Interplay and Predictors of Missed Diagnostic Opportunities

**Figure 1:**
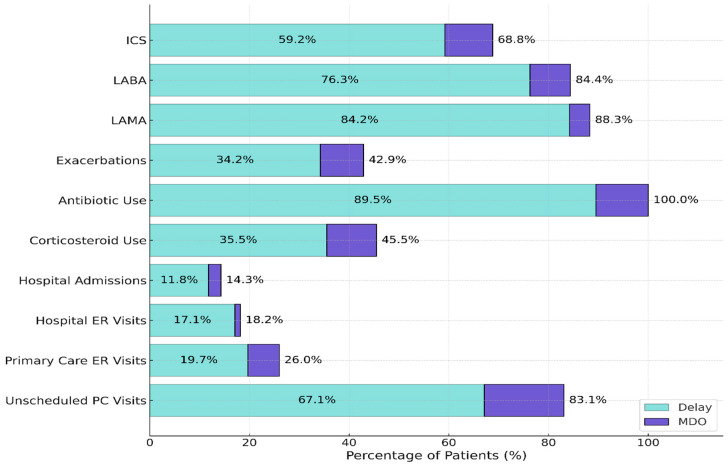
Overlap of healthcare utilization and treatment indicators between patients with diagnostic delay and missed diagnostic opportunities (MDOs).

To investigate the relationship between diagnostic delay and missed opportunities, the overlap between these two phenomena was first examined. A substantial co-occurrence was observed: a total of 87.3% of patients who met the criteria for MDO also experienced a diagnostic delay (69 of 79 patients). Conversely, only 10 patients (12.7%) were classified as MDO-positive despite having no documented delay. Figure 1 provides a detailed visualization of this overlap across specific pre-diagnostic healthcare utilization and treatment indicators. Among patients with diagnostic delay, the most frequent events were antibiotic use (89.5%), LAMA initiation (84.2%), and unscheduled primary care visits (67.1%), while events such as hospital admissions were less common (11.8%). A similar pattern, with consistently higher percentages across most indicators, was observed in the MDO group.

The bars represent the percentage of patients who experienced each healthcare utilization or treatment indicator in the 12 months prior to diagnosis. Darker sections reflect patients classified as MDO-positive, while lighter sections represent patients with diagnostic delay. All values are presented descriptively to avoid inferential bias, as several indicators are components of the composite MDO definition. ICS: Inhaled Corticosteroid; LABA: Long-Acting Beta-Agonist; LAMA: Long-Acting Muscarinic Antagonist; ER: Emergency Room; PC: Primary Care.

To determine if diagnostic delay was an independent predictor of MDO, a multivariable logistic regression analysis was performed. After adjusting for baseline clinical severity indicators—including symptom burden, airflow limitation, and exacerbation frequency—diagnostic delay remained a strong and statistically significant predictor of having missed diagnostic opportunities (OR 10.25, 95% CI [4.39, 24.88]; *p* < 0.001). The full model details are presented in Table 3.

### 3.3. Escalating Clinical Burden and Identification of Patient Phenotypes

**Figure 2:**
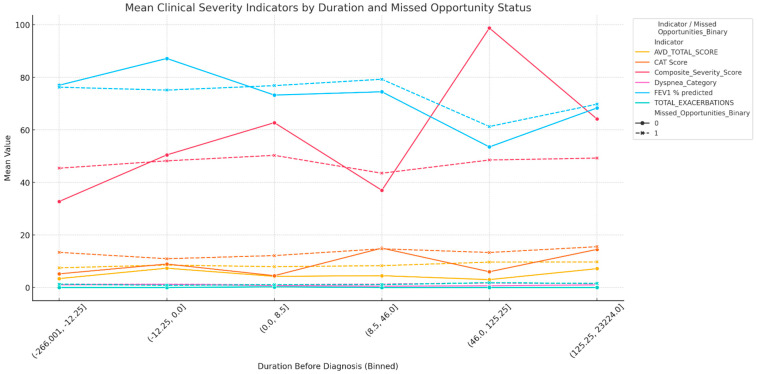
Patterns of clinical severity indicators across diagnostic delay intervals, stratified by missed diagnostic opportunity status. Note Figure 2: Mean values of six clinical severity indicators are plotted across binned intervals of diagnostic delay. Patients are stratified by the presence (missed) or absence (not missed) of missed diagnostic opportunities. Delay bins reflect increasing time from the first healthcare contact to the final COPD diagnosis. AVD: Activity of Daily Living; CAT: COPD Assessment Test; FEV_1_: Forced Expiratory Volume in 1 s.

To further explore the dynamics of diagnostic delay, the relationship between the duration of the delay and clinical severity was analyzed. A clear dose–response pattern was observed, where a longer pre-diagnostic interval was associated with a progressively greater clinical burden, particularly among patients with missed diagnostic opportunities (Figure 2). In this MDO-positive group, the mean number of total exacerbations rose from 0.5 in the shortest delay interval to 3.5 in the longest (*p* <0.001). Similarly, CAT scores increased more sharply in the MDO-positive group (from a mean of 10.3 to 16.1) compared to the MDO-negative group (from 7.4 to 10.0), with significant between-group differences (*p* < 0.001). Dyspnea severity also increased significantly in the MDO-positive group (from a mean mMRC of 1.2 to 2.1, *p* = 0.014), while FEV_1_ % predicted declined from 78.3% to 61.2%, a steeper drop than that observed in the non-missed group (76.5% to 70.4%). In contrast, the composite severity score showed minimal separation between groups and no statistically significant changes across delay intervals (*p* = 0.420).

To identify underlying patient profiles that might explain these different diagnostic trajectories, an unsupervised K-means cluster analysis was performed. This analysis revealed three distinct clinical phenotypes: “Paucisymptomatic-–Preserved” (n = 73), “Frequent Attenders/High-Risk” (n = 64), and “Silent Decliners” (n = 30). As detailed in Table 4, the “Frequent Attenders/High-Risk” group was characterized by severe airflow obstruction, the highest symptom burden, and a markedly elevated frequency of pre-diagnostic exacerbations. In contrast, the “Paucisymptomatic–Preserved” group presented with relatively preserved lung function and minimal symptom burden, yet exhibited a high rate of diagnostic delay. The Silent Decliners showed severe airflow limitation despite only moderate symptom expression and relatively low healthcare utilization prior to diagnosis.

### 3.4. Multidimensional Risk Profiles and Predictive Stratification

Finally, to validate the clinical utility of the predictive model and synthesize the study’s findings, patients were stratified into three risk groups (low, moderate, and high) based on their predicted probability of diagnostic delay. As shown in Table 5, this stratification effectively discriminated patients, revealing a clear gradient of clinical severity. The high-risk group exhibited significantly worse airflow limitation, greater symptom burden, and higher rates of healthcare utilization and treatment prescription compared to the low- and moderate-risk groups.

**Figure 3:**
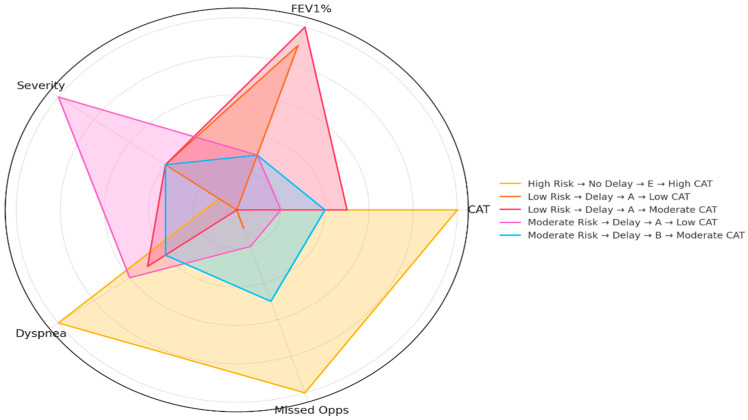
Multidimensional clinical profiles across the top five most frequent diagnostic patterns. The radar chart presents normalized values (0–1 range) for five clinical domains. Each line represents one of the top five most common diagnostic patterns, which combine predicted risk group, diagnostic delay status, GOLD classification, and CAT score tertile. This allows for a visual comparison of the multidimensional clinical burden across distinct patient profiles. CAT: COPD Assessment Test; FEV_1_: Forced Expiratory Volume in 1 s.

To visualize the interplay of these multiple dimensions, the five most frequent diagnostic patterns observed in the cohort were characterized (Figure 3). The most clinically severe profile was “High Risk → No Delay → E → High CAT” (n = 8), defined by a CAT score of 18.5 (SD = 2.73), FEV_1_ % of 59.4 (SD = 22.4), an mMRC score of 1.62 (SD = 0.92), and a missed opportunities score of 569.1 (SD = 53.3). In contrast, the “Low Risk → Delay → A → Low CAT” group (n = 26) traced the smallest polygon, with a CAT score of 5.69 (SD = 2.31), FEV_1_ % of 90.4 (SD = 14.8), and an mMRC score of 0.81 (SD = 0.57). Two additional patterns reflected intermediate severity: the “Low Risk → Delay → A → Moderate CAT” group (n = 12) and the “Moderate Risk → Delay → B → Moderate CAT” group (n = 10). Statistical comparisons across these five patterns were significant for CAT score (H(4) = 57.29, *p* < 0.001), FEV_1_ % predicted (H(4) = 32.68, *p* < 0.001), dyspnea severity (H(4) = 12.51, *p* = 0.0139), and missed opportunities (H(4) = 45.89, *p* < 0.001). The composite severity score did not differ significantly (H(4) = 1.95, *p* = 0.745).

## 4. Discussion

This study provides a multidimensional analysis of the pre-diagnostic phase of COPD in a real-world primary care setting, demonstrating that diagnostic delay and missed diagnostic opportunities (MDOs) are not only prevalent but are also intrinsically linked and associated with a substantially greater clinical burden at the time of diagnosis. Our principal findings reveal that diagnostic delay is a powerful independent predictor of MDOs, that the clinical burden escalates in a dose-dependent manner with the duration of this delay, and that patients follow distinct, identifiable clinical phenotypes on their journey to diagnosis. These findings challenge the notion of a passive waiting period and reframe the pre-diagnostic phase as a dynamic process of detectable, and therefore potentially preventable, clinical deterioration.

### 4.1. The Interplay of Diagnostic Delay and Missed Opportunities

A central finding of this study is the profound interplay between diagnostic delay and MDOs. While previous research has highlighted the high frequency of missed opportunities in the years preceding a COPD diagnosis [22], our work moves beyond description to establish a strong, independent predictive link. The multivariable analysis (Table 3) showed that a delay of over 30 days increased the odds of a patient accumulating a significant burden of MDOs more than tenfold, even after adjusting for baseline clinical severity. This suggests that diagnostic delay is not merely a consequence of missed opportunities, but a powerful independent predictor of them, creating a vicious cycle of clinical inertia [23]. The substantial overlap observed, where nearly 87.3% of MDO-positive patients also experienced a delay (Figure 1), reinforces that these are two facets of the same systemic issue: a failure to act on accumulating clinical signals [24]. The shared pattern of healthcare interactions across both groups further supports this convergence. As illustrated in Figure 1, high percentages of patients with diagnostic delay and MDOs had prior unscheduled visits, emergency encounters, and early treatment initiations—especially inhaled corticosteroids and antibiotics—suggesting that diagnostic inertia occurred despite clear clinical triggers. These findings highlight systemic inefficiencies in recognizing and responding to escalating respiratory symptoms, particularly in primary care.

Our data, derived from a primary care cohort where patients had frequent healthcare interactions (Table 1), contradict the assumption that delays are primarily due to patients not seeking care [25]. Instead, it points towards a diagnostic inertia within the healthcare system, a phenomenon increasingly recognized in chronic disease management [26]. The fact that patients with delay already presented with worse lung function and higher symptom burden (Table 1) indicates that the signals were present but not acted upon, a critical distinction from truly subclinical disease. This quantifies the phenomenon of diagnostic inertia—a failure to initiate or intensify diagnostic action despite the presence of clear indicators—and establishes it as a major barrier in the primary care of COPD.

### 4.2. Escalating Clinical Burden and Novel Patient Phenotypes

This study uniquely demonstrates that the pre-diagnostic period is not clinically static. The dose–response relationship illustrated in Figure 2 reveals a clear pattern of escalating clinical severity—particularly in exacerbations and symptom scores—that correlates with the duration of the diagnostic delay. This finding provides a crucial temporal dimension to previous cross-sectional reports linking delayed diagnosis to poorer outcomes [27] and suggests that inaction allows for preventable disease progression. This observation is consistent with evidence suggesting that the rate of FEV_1_ decline is, in fact, most rapid during the early-to-moderate stages of COPD, providing a strong biological plausibility for why this pre-diagnostic period represents such a critical window for intervention [28].

The identification of three distinct clinical phenotypes via cluster analysis (Table 4) offers a novel, data-driven framework for understanding why these different trajectories occur. The “Frequent Attenders/High-Risk” group represents a clinical paradox: despite high healthcare utilization and severe underlying disease, they still accumulate MDOs. This points towards issues of care fragmentation or a failure to synthesize longitudinal data in busy clinical settings, a recognized barrier to optimal chronic care [29]. Conversely, the “Silent Decliners” phenotype, characterized by severe airflow obstruction but lower symptom expression, highlights the inherent limitations of purely symptom-driven diagnostic strategies and underscores the urgent need for proactive case-finding in high-risk individuals [30]. Finally, the “Paucisymptomatic–Preserved” group demonstrated preserved lung function and low symptom burden, yet paradoxically exhibited high rates of diagnostic delay. This pattern suggests that patients perceived as clinically stable may be systematically deprioritized in diagnostic workflows, reinforcing the need to move beyond symptom-based case-finding in primary care [31].

### 4.3. Clinical Implications and a Shift Towards Proactive Stratification

The findings of this study have significant implications for primary care. The traditional, reactive approach to diagnosis—often triggered by an acute event—is clearly insufficient. Our results advocate for a paradigm shift towards proactive, data-driven risk stratification. The validation of our risk prediction model (Table 5) demonstrates that it is possible to identify patients at high risk for diagnostic delay using routinely collected clinical data. This creates an opportunity for targeted interventions, such as automated EMR-based alerts or prioritized spirometry for patients matching a high-risk profile, moving beyond simple screening questionnaires [32].

Furthermore, the multidimensional patient profiles visualized in the radar chart (Figure 3) suggest that a “one-size-fits-all” case-finding strategy is likely to fail. Interventions may need to be tailored to the specific phenotypes identified. For instance, “Frequent Attenders” may benefit from integrated care pathways and data synthesis tools that flag accumulating risk over time, while “Silent Decliners” may only be identified through systematic screening of at-risk populations based on age and smoking history [33]. This personalized approach to diagnostics aligns with the broader move towards precision medicine in chronic disease management [34]. Crucially, this approach moves beyond traditional case-finding based on static risk factors towards a more dynamic, pre-diagnostic phenotyping, which may be essential for targeting the right intervention to the right patient profile.

### 4.4. Strengths and Limitations

This study has several strengths, including its use of detailed, longitudinal real-world data from a primary care setting, the application of a novel, integrative, analytical approach combining predictive modeling and unsupervised clustering, and the development of robust, data-driven operational definitions for key outcomes. However, several limitations must be acknowledged. First, its retrospective design is susceptible to unmeasured confounding and relies on the accuracy of clinical documentation. While we focused on objective events, the rationale behind clinical decisions remains uncaptured. However, we mitigated this by focusing on objective, verifiable events such as prescriptions and hospital admissions, which are less susceptible to documentation bias. Second, the findings are from a single healthcare system in Spain, and while the observed patterns are consistent with the international literature [35], generalizability to systems with different structures or access to spirometry requires confirmation. The Spanish National Health System offers universal coverage and is structured around a strong primary care gatekeeper model that ensures high care continuity. As reflected by the frequent healthcare interactions in our cohort, patients had high access to care. While spirometry is generally available in primary care settings, its implementation can be variable. The fact that significant diagnostic inertia occurred within a system with these features suggests that the problem is not merely a lack of resources or access, but rather a more fundamental failure to act on accumulating clinical triggers. This implies our findings are relevant to other healthcare environments, as similar challenges with clinical inertia likely exist regardless of the system’s structure and may even be exacerbated in settings with greater barriers to care. Nevertheless, the clinical phenotypes identified are consistent with patient journeys described in the international qualitative literature, suggesting a degree of external validity to our findings. Third, our operational definition of diagnostic delay, while internally validated and effective for risk stratification in this cohort, is not a universally established standard. However, it proved to be a clinically relevant and effective tool for risk stratification within this cohort, successfully discriminating patients with different outcomes. Fourth, our study focused on the diagnostic pathway within primary care and did not include systematic data on pulmonologist referral or involvement. This is an important consideration, as timely access to specialist care has been shown to improve patient outcomes [36]. Fifth, while we excluded patients with other chronic respiratory diseases, we did not perform a systematic analysis of non-respiratory comorbidities (e.g., cardiovascular disease, anxiety), which could undoubtedly influence symptom perception and healthcare utilization, potentially contributing to diagnostic delay. Finally, the predictive models and clinical phenotypes identified require prospective validation in independent cohorts before they can be implemented in clinical practice [37]. Despite these limitations, this study provides a robust and granular characterization of the pre-diagnostic journey, offering a strong, evidence-based foundation for the design of future prospective and interventional research.

## 5. Conclusions

This study demonstrates that the pre-diagnostic period in COPD is not a passive waiting time but a dynamic window of detectable, and potentially preventable, clinical deterioration. By establishing that diagnostic delay is a powerful driver of missed opportunities and is associated with an escalating clinical burden, our findings quantify the tangible impact of diagnostic inertia in primary care.

The identification of distinct clinical phenotypes—the “Frequent Attender/High-Risk,” the “Paucisymptomatic–Preserved,” and the “Silent Decliner”—provides a novel, data-driven framework to move beyond a one-size-fits-all approach to case-finding. Ultimately, our results provide a robust evidence base for shifting the diagnostic paradigm from reactive management towards a proactive, stratified, and personalized approach, where data-driven tools can help identify high-risk patients before the irreversible consequences of a delayed diagnosis take hold.

## Figures and Tables

**Table 1:** Comparison of clinical, functional, and healthcare characteristics by diagnostic delay status.

Variable	Delay (n = 76)	No Delay (n = 91)	*p*-Value
**Demographics**			
Age (years)	60.24 ± 20.75	64.93 ± 17.30	0.1213
Weight (kg)	77.77 ± 17.31	72.73 ± 13.36	0.0402
Height (cm)	165.17 ± 11.33	164.75 ± 7.83	0.7849
BMI (kg/m^2^)	28.59 ± 6.47	26.87 ± 5.11	0.0641
Pack-years	36.82 ± 19.78	34.29 ± 12.53	0.3456
**Pulmonary Function**			
FEV_1_ (mL)	1686.4 ± 647.4	1961.4 ± 607.7	0.0368
FVC (mL)	2749.6 ± 1054.2	2985.1 ± 960.7	0.0034
FEV_1_/FVC ratio	62.38 ± 7.49	61.26 ± 8.52	0.5584
**GOLD Classification**			0.0623
A	21 (27.6%)	31 (34.1%)	
B	32 (42.1%)	37 (40.7%)	
E	23 (30.3%)	23 (25.3%)	
**Respiratory Symptoms and Burden**			
CAT Score	13.57 ± 6.13	10.62 ± 6.06	0.0006
CAT ≥ 10	89 (66.4%)	20 (62.5%)	0.0731
Dyspnea ≥ 2 (mMRC)	57 (42.5%)	15 (46.9%)	0.0565
Total Exacerbations, m (SD)	1.66 ± 2.15	0.31 ± 0.92	<0.001
Chronic Cough	105 (78.4%)	25 (78.1%)	0.0581
AVD Total Score	8.72 ± 4.07	7.44 ± 4.01	0.0563
GesEPOC Risk			0.0075
High	48 (63%)	37 (41%)	
Low	28 (37%)	54 (59.3%)	
**Physical Activity**			
IPAQ (MET-min/week)	2345.24 ± 7365.01	1592.15 ± 3119.64	0.3519
Total_MET_minutes	4166.78 ± 6845.32	3351.47 ± 5980.11	0.4225
**Treatments**			
LABA	43 (32.1%)	6 (18.8%)	0.0261
LAMA	64 (84.2%)	65 (71.4%)	0.0579
ICS	45 (59.2%)	38 (41.8%)	0.0367
**Healthcare Utilization**			
Unscheduled PC Visits	2.51 ± 2.21	1.32 ± 1.31	<0.001
Primary Care ER Visits	0.92 ± 1.24	0.20 ± 0.60	<0.001
Hospital ER Visits	0.59 ± 0.95	0.09 ± 0.32	<0.001
Hospital Admissions	0.14 ± 0.42	0.02 ± 0.15	0.0129
Pulmonary function test performed at first symptomatic visit, n (%)	65 (85.5%)	91 (100%)	<0.001
Delay (Days)	128.5 [60.0–234.2]	13.0 [8.0–20.0]	N/A

Data are presented as mean ± SD, median [interquartile range], or n (%). *p*-values were calculated using independent-sample *t*-tests or Mann–Whitney U tests for continuous variables, and Chi-square or Fisher’s exact tests for categorical variables. AVD: Activity of Daily Living; BMI: Body Mass Index; CAT: COPD Assessment Test; ER: Emergency Room; FEV_1_: Forced Expiratory Volume in 1 s; FVC: Forced Vital Capacity; GesEPOC: Spanish COPD Guideline; GOLD: Global Initiative for Chronic Obstructive Lung Disease; ICS: Inhaled Corticosteroid; IPAQ: International Physical Activity Questionnaire; LABA: Long-Acting Beta-Agonist; LAMA: Long-Acting Muscarinic Antagonist; MET: Metabolic Equivalent of Task; mMRC: modified Medical Research Council Dyspnea Scale; PC: Primary Care; N/A: Not applicable.

**Table 2:** Comparison of clinical, functional, and healthcare characteristics by missed diagnostic opportunity status.

Variable	MDO (n = 79)	No MDO (n = 88)	*p*-Value
**Demographics**			
Age (years)	61.91 ± 21.18	64.20 ± 16.94	0.3197
Weight (kg)	76.90 ± 15.54	73.43 ± 15.28	0.1500
Height (cm)	165.92 ± 8.89	164.10 ± 10.08	0.2166
BMI (kg/m^2^)	27.91 ± 5.36	27.44 ± 6.21	0.5986
Pack-years	34.35 ± 17.15	36.35 ± 15.37	0.3456
Current smokers (%)	44 (55.8%)	51 (58.4%)	0.7482
Pulmonary Function			
FEV_1_ (mL)	1577.0± 645.6	1897.9 ± 611.1	0.0080
FVC (mL)	2591.3± 1060.9	2801.9 ± 1162.3	0.0010
FEV_1_/FVC ratio	61.83 ± 8.32	62.08 ± 8.02	0.3230
GOLD Classification			0.0331
A	21 (26.6%)	32 (36.4%)	
B	32 (40.5%)	42 (47.7%)	
E	26 (32.9%)	14 (15.9%)	
**Respiratory Symptoms and Burden**			
CAT Score	13.92 ± 6.20	10.43 ± 6.22	0.0008
CAT ≥ 10	84 (64.6%)	25 (69.4%)	0.0723
Dyspnea ≥ 2 (mMRC)	59 (45.4%)	13 (36.1%)	0.0605
Total exacerbations	1.24 ± 1.86	0.71 ± 1.31	0.0323
Chronic cough (%)	101 (77.7%)	29 (80.6%)	0.0046
AVD Total Score	8.26 ± 4.10	7.50 ± 4.06	0.2054
GesEPOC high-risk profile	70 (53.8%)	15 (41.7%)	0.0100
**Physical Activity**			
IPAQ (MET-min/week)	1974.88 ± 5628.20	2043.78 ± 5096.62	0.9143
Total MET minutes	3873.80 ± 6453.00	3963.55 ± 6406.22	0.9124
**Respiratory Treatments**			
LABA	39 (30.0%)	10 (27.8%)	0.0451
LAMA	68 (86.1%)	61 (69.3%)	0.0070
ICS	53 (67.1%)	30 (34.1%)	<0.001
**Healthcare Utilization**			
Unscheduled PC visits	2.26 ± 2.15	1.58 ± 1.69	NA
Primary Care ER visits	0.80 ± 1.13	0.37 ± 0.85	NA
Hospital ER visits	0.41 ± 0.80	0.18 ± 0.50	NA
Hospital admissions	0.11 ± 0.34	0.04 ± 0.21	NA
Delay (Days)	89.0 [23.0–201.0]	16.0 [9.0–34.0]	<0.001

Data are presented as mean ± SD, median [interquartile range], or n (%). *p*-values were calculated using independent-sample *t*-tests or Mann–Whitney U tests for continuous variables, and Chi-square or Fisher’s exact tests for categorical variables. Inferential testing was not performed for healthcare utilization variables or delay (days) as these indicators contribute to the MDO score definition. AVD: Activity of Daily Living; BMI: Body Mass Index; CAT: COPD Assessment Test; ER: Emergency Room; FEV_1_: Forced Expiratory Volume in 1 s; FVC: Forced Vital Capacity; GesEPOC: Spanish COPD Guideline; GOLD: Global Initiative for Chronic Obstructive Lung Disease; ICS: Inhaled Corticosteroid; IPAQ: International Physical Activity Questionnaire; LABA: Long-Acting Beta-Agonist; LAMA: Long-Acting Muscarinic Antagonist; MDO: Missed Diagnostic Opportunity; MET: Metabolic Equivalent of Task; mMRC: modified Medical Research Council Dyspnea Scale; PC: Primary Care.

**Table 3:** Multivariable logistic regression analysis of the association between diagnostic delay and missed diagnostic opportunities. The dependent variable for all models was the binary MDO status (≥4 points = positive).

Model	Predictor	B	SE	Pseudo R^2^	AIC	OR	*p*-Value	95% CI for OR	RRI
Model 1: Diagnostic Delay (Binary)	Diagnostic Delay (Yes)	2.779	0.389	0.278	169.46	16.1	<0.001	[7.51, 34.55]	1510%
Model 2: Diagnostic Delay (Days)	Delay (Days)	0.009	0.002	0.191	189.41	1.01	<0.001	[1.01, 1.01]	1%
Model 3: Clinical Predictors Only	CAT Score	−0.059	0.053	0.536	117.71	0.94	0.270	[0.85, 1.05]	−6%
	FEV_1_ % predicted	−0.017	0.014	—	—	0.98	0.211	[0.96, 1.01]	−2%
	AVD Score	0.105	0.079	—	—	1.11	0.186	[0.95, 1.30]	11%
	Total Exacerbations	0.206	0.147	—	—	1.23	0.162	[0.93, 1.63]	23%
	Dyspnea (mMRC ≥ 2)	0.245	0.214	—	—	1.28	0.253	[0.83, 1.97]	28%
Model 4: Delay + Clinical Predictors	Diagnostic Delay (Yes)	2.327	0.485	0.613	102.34	10.25	<0.001	[4.39, 24.88]	925%
	CAT Score	−0.039	0.061	—	—	0.96	0.522	[0.85, 1.09]	−4%
	FEV_1_ % predicted	−0.015	0.014	—	—	0.99	0.254	[0.96, 1.02]	−1%
	AVD Score	0.051	0.083	—	—	1.05	0.542	[0.89, 1.24]	5%
	Total Exacerbations	0.262	0.158	—	—	1.30	0.097	[0.95, 1.78]	30%
	Dyspnea (mMRC ≥ 2)	0.312	0.228	—	—	1.37	0.172	[0.87, 2.15]	37%

Model 1 includes only the binary diagnostic delay variable. Model 2 includes delay as a continuous variable. Model 3 includes only clinical predictors. Model 4 is the full model adjusting for both binary delay and clinical predictors. AIC: Akaike Information Criterion; AVD: Activity of Daily Living; B: Unstandardized logistic regression coefficient; CAT: COPD Assessment Test; CI: Confidence Interval; FEV_1_: Forced Expiratory Volume in 1 s; mMRC: modified Medical Research Council Dyspnea Scale; OR: Odds Ratio; SE: Standard Error.

**Table 4:** Clinical and healthcare utilization characteristics of patient phenotypes identified by clustering.

Variable	Paucisymptomatic–Preserved (n = 73)	Frequent Attenders/High-Risk (n = 64)	Silent Decliners (n = 30)	*d* HS– FA	*d* HS– SD	*d* SD– FA
Age (years)	64.94 ± 16.28	64.07 ± 16.25	59.83 ± 22.63	0.05	0.28	0.23
BMI (kg/m^2^)	27.23 ± 4.73	28.55 ± 6.28	27.72 ± 6.69	−0.24	−0.09	0.13
Pack-years	33.14 ± 13.71	40.00 ± 20.54	35.87 ± 16.31	−0.40	−0.19	0.21
FEV_1_ % predicted	86.20 ± 15.50	69.21 ± 18.70	64.06 ± 15.84	1.00	1.42	0.29
CAT Score	7.44 ± 3.09	17.20 ± 4.76	14.61 ± 6.13	−2.47	−1.71	0.50
AVD Total Score	5.52 ± 2.93	10.53 ± 3.93	9.70 ± 3.82	−1.46	−1.30	0.21
Composite Severity Score	47.06 ± 26.11	52.85 ± 27.12	49.84 ± 31.19	−0.22	−0.10	0.11
Dyspnea (mMRC)	1.14 ± 0.76	1.43 ± 0.73	1.31 ± 0.73	−0.39	−0.23	0.16
Total Exacerbations	0.12 ± 0.33	4.00 ± 2.03	0.39 ± 0.58	−2.76	−0.65	2.11
Sex (Female)	32 (43.84%)	15 (23.44%)	29 (96.66%)	0.44	−0.03	−0.47
Missed Opportunities Binary (Yes)	45 (61.64%)	60 (93.75%)	26 (86.66%)	−0.83	−0.57	0.26
Diagnostic Delay > 30 days	59 (80.82%)	15 (23.44%)	27 (90.0%)	1.22	0.28	−0.94
Delay_(Days)	23.0 [12.0–83.0]	171.5 [73.2–285.5]	30.0 [16.0–259.2]	−1.30	−0.07	0.86
IPAQ Activity Level: Low	12 (16.44%)	18 (28.13%)	9 (30.0%)	0.07	−0.21	−0.28
IPAQ Activity Level: Moderate	30 (41.10%)	24 (37.50%)	7 (23.33%)	0.50	0.54	0.04
IPAQ Activity Level: High	31 (42.47%)	22 (34.38%)	14 (46.66%)	0.65	0.34	−0.31

Data are presented as mean ± SD or n (%). Between-group differences were quantified using standardized effect sizes (Cohen’s d for continuous variables and Cohen’s h for categorical proportions) to avoid inferential circularity, as these variables were used in the clustering algorithm. AVD: Activity of Daily Living; BMI: Body Mass Index; CAT: COPD Assessment Test; FEV_1_: Forced Expiratory Volume in 1 s; IPAQ: International Physical Activity Questionnaire; mMRC: modified Medical Research Council Dyspnea Scale.

**Table 5:** Baseline clinical characteristics stratified by predicted risk group.

Variable	Low Risk (n = 56)	Moderate Risk (n = 54)	High Risk (n = 57)	*p*-Value
Age (years)	67.8 ± 12.83	60.81 ± 20.03	59.62 ± 22.19	<0.001
BMI (kg/m^2^)	27.43 ± 5.15	27.91 ± 5.07	27.65 ± 7.12	0.752
Pack-years	32.12 ± 12.51	32.25 ± 12.42	41.76 ± 20.66	0.035
FEV_1_ % predicted	71.99 ± 13.81	74.45 ± 11.37	57.65 ± 14.20	<0.001
FVC % predicted	97.94 ± 15.00	85.13 ± 15.22	73.08 ± 17.08	<0.001
FEV_1_/FVC ratio	0.67 ± 0.09	0.64 ± 0.09	0.63 ± 0.09	0.001
CAT Score	8.27 ± 3.92	9.83 ± 4.68	13.07 ± 6.24	<0.001
AVD Score	3.31 ± 1.67	3.57 ± 2.15	4.91 ± 2.65	<0.001
IPAQ—METs	3382.13 ± 2577.57	2252.21 ± 1689.64	1692.13 ± 1388.00	<0.001
Unscheduled Primary Care Visits	2.62 ± 1.94	3.28 ± 2.15	4.29 ± 2.83	0.010
Primary Care ER Visits	0.60 ± 0.82	0.96 ± 0.91	1.39 ± 1.15	0.002
Hospital ER Visits	0.36 ± 0.61	0.62 ± 0.91	1.09 ± 1.04	<0.001
Hospital Admissions	0.13 ± 0.34	0.22 ± 0.44	0.44 ± 0.62	0.019
Total Exacerbations	0.58 ± 0.83	1.01 ± 1.10	1.84 ± 1.36	<0.001
Composite Severity Score	26.93 ± 18.23	39.17 ± 18.70	53.83 ± 21.83	0.316
Inhaled Maintenance Treatment	36 (64.29%)	49 (90.74%)	54 (94.74%)	<0.001
Systemic Corticosteroids (≥1/year)	5 (8.93%)	13 (24.07%)	22 (38.60%)	<0.001
Antibiotic Courses (≥1/year)	11 (19.64%)	19 (35.19%)	33 (57.89%)	<0.001
LABA	19 (33.93%)	44 (81.48%)	53 (92.98%)	<0.001
LAMA	18 (32.14%)	45 (83.33%)	54 (96.4%)	<0.001
ICS	2 (3.6%)	15 (27.77%)	29 (50.88%)	<0.001
Dyspnea ≥ 2 (mMRC)	15 (26.79%)	18 (33.33%)	23 (40.36%)	0.0776

Data are presented as mean ± SD or n (%). Risk groups were derived from tertiles of the predicted probability of diagnostic delay from a multivariable logistic regression model. Statistical comparisons were performed using ANOVA or Chi-square tests as appropriate. AVD: Activity of Daily Living; BMI: Body Mass Index; CAT: COPD Assessment Test; ER: Emergency Room; FEV_1_: Forced Expiratory Volume in 1 s; FVC: Forced Vital Capacity; ICS: Inhaled Corticosteroid; IPAQ: International Physical Activity Questionnaire; LABA: Long-Acting Beta-Agonist; LAMA: Long-Acting Muscarinic Antagonist; METs: Metabolic Equivalent Tasks; mMRC: modified Medical Research Council Dyspnea Scale.

## Data Availability

The dataset is available on request from the authors.

## Supplementary Materials

The following supporting information can be downloaded at: https://www.mdpi.com/article/10.3390/diagnostics15172209/s1, Figure S1: Study Flow Diagram (STROBE).

## Abbreviations

The following abbreviations are used in this manuscript:

PC	Primary Care
GOLD	Global Initiative for Chronic Obstructive Lung Disease
BMI	Body mass index
CAT	COPD Assessment Test score
mMRC	Modified Medical Research Council Dyspnea Scale
GesEPOC	Spanish COPD guidelines
ER	Emergency room
AVD	Activity Limitation Score
IPAQ	International Physical Activity Questionnaire
MET	Metabolic equivalent minutes
MDO	Missed Diagnostic Opportunities
AIC	Akaike Information Criterion
BIC	Bayesian Information Criterion
AUC	Area under the curve
VIF	Variance inflation factors
PCA	Principal component analysis

## Appendix A. Methods: Assessment of Multicollinearity

To assess the stability of the multivariable logistic regression models, multicollinearity was evaluated using Variance Inflation Factor (VIF) calculations for all predictors. The results are presented below.

**Table A1:** Variance Inflation Factors (VIFs) for Multivariable Model Predictors. VIF calculations were performed on all clinically relevant predictors considered for the final multivariable model to ensure the absence of collinearity among candidate variables.

Variable	VIF
Diagnostic Delay (Yes/No)	1.65
CAT Score	1.82
FEV_1_ % predicted	1.74
AVD Score	1.58
Dyspnea (mMRC ≥ 2)	1.27
Total Exacerbations	1.36
Systemic Corticosteroid Use (Yes/No)	1.48
Systemic Antibiotic Use (Yes/No)	2.72
Inhaled Maintenance Treatment (Yes/No)	2.21
Missed Diagnostic Opportunity (Binary)	2.69

No predictor exhibited VIF values suggestive of problematic collinearity (VIF > 5).

Statistical Power and Sample Size

A priori power analysis was conducted to determine the minimum sample size required to detect a statistically significant association between diagnostic delay (>30 days) and clinical predictors using logistic regression. Assuming a medium effect size (odds ratio = 1.5), 80% power, and a two-tailed alpha of 0.05, the estimated required sample size was approximately n = 148. The final analytic cohort (n = 167) exceeded this threshold, allowing for sufficient statistical power and subgroup comparisons.

Post hoc evaluation confirmed the adequacy of the sample. With 76 patients having the primary outcome (experiencing diagnostic delay) and 6 predictors included in the final multivariable model, the events-per-variable (EPV) ratio was 12.7—well above the conventional minimum of 10 EPV recommended to ensure model stability. This finding supports the robustness of the sample size for the planned analyses.

**Table A2:** Sample size and statistical power assessment. Power calculations assumed a logistic regression framework with a binary outcome (delay > 30 days). Post hoc power estimation used simulation-based approximation for multivariable models.

Parameter	Value
Total cohort size (N)	167
Diagnostic delay events (n, %)	76 (45.5%)
Number of predictors in final model	6
Events per variable (EPV)	12.7
Minimum recommended EPV	10
A priori estimated sample size (OR = 1.5, 80% power, α = 0.05)	~150
Estimated post hoc statistical power	>80%

Formula used (Logistic Regression Power Estimation): For a binary outcome with logistic regression, sample size estimation can be approximated by n = (Z1 − α/2 + Z1 − β)^2^/p(1 − p)(ln(OR))^2^

where

- n = required sample size per group;
- Z1 − α/2 = Z-score for two-tailed significance level (e.g., 1.96 for α = 0.05);
- Z1 − β = Z-score for desired power (e.g., 0.84 for 80% power);
- *p* = proportion of cases in the reference group (e.g., *p* = 0.5 or estimated from data);
- OR = expected odds ratio;
- ln(OR) = natural log of the odds ratio.

This formula is an approximation and assumes a balanced design; in real-world studies, more complex simulations or software tools (e.g., G*Power, R pwr package, version 1.3-0) are often used to refine estimates.

Sensitivity Analyses for Diagnostic Delay Threshold

To test the robustness of the definition of diagnostic delay, alternative thresholds were examined (30, 60, 90, and 120 days). The strength and significance of associations with missed diagnostic opportunities remained consistent across all thresholds.

**Table A3:** Sensitivity analysis: alternative diagnostic delay thresholds. Higher effect sizes (r) and lower *p*-values support stronger separation between groups. The >30-day threshold demonstrated the strongest effect for missed diagnostic opportunities and clinical severity. Mann–Whitney U tests were used; r is calculated as Z/√N.

Delay Threshold	Outcome	U Statistic	*p*-Value	Effect Size (r)
>30 days	Missed Diagnostic Opportunities	584	<0.001	0.67
	Composite Severity Score	935.5	0.420	0.52
>60 days	Missed Diagnostic Opportunities	673	<0.001	0.61
	Composite Severity Score	1012	0.420	0.47
>90 days	Missed Diagnostic Opportunities	701.5	<0.001	0.58
	Composite Severity Score	1046	0.420	0.43
>120 days	Missed Diagnostic Opportunities	750	<0.001	0.53
	Composite Severity Score	1075.5	0.420	0.39

The findings support the use of ≥30 days as a clinically meaningful and statistically robust cutoff for defining diagnostic delay, balancing statistical significance, clinical plausibility, and subgroup sample size, despite maintained associations at higher thresholds.

**Table A4:** Sensitivity analysis of diagnostic delay classification using proxy indicators of acute-onset presentation.

Group	N	Delay (n)	Delay (%)	χ^2^	*p*-Value
Full Sample	167	76	45.5	—	—
Excluding ER-based Index Visit	149	69	46.3	1.26	0.888
Excluding Steroid/Antibiotic Index	150	69	46.0	3.14	0.914
Excluding Any Exacerbation Proxy	138	66	46.7	2.48	0.711

To evaluate whether the classification of diagnostic delay was biased by the acuity of the initial clinical presentation, we conducted a sensitivity analysis excluding patients with proxy indicators of exacerbation-related care: (1) emergency room (ER) visits and (2) prior use of systemic corticosteroids or antibiotics. A combined exclusion was also tested. The proportion of diagnostic delay remained unchanged across all sensitivity scenarios. These findings suggest that the observed diagnostic delay is not driven by acute onset encounters, but rather reflects broader patterns of clinical inertia and underdiagnosis.

Cluster Validation Procedures

Cluster analysis was conducted to identify diagnostic trajectories and patient profiles. The optimal number of clusters was determined through three complementary validation methods.

**Table A5:** **Panel A.** Cluster validation metrics.

Criterion	Optimal Number of Clusters	Justification
Elbow Method	3	Inflection point observed
Silhouette Score	3	Maximum silhouette coefficient
Davies-Bouldin Index	3	Lowest index value

All metrics supported the selection of three clusters, reinforcing the validity of the trajectory classification used.

**Panel B**. Kruskal–Wallis test across diagnostic delay duration bins.

**Indicator**	**H (df = 9)**	***p*****-Value**
Frequent PreDx Use	46.37	<0.001
Significant Dyspnea	34.28	<0.001
Frequent Exacerbations	39.14	<0.001
Frequent Treatment Exposure	45.91	<0.001
CAT Score	14.865	0.0019
AVD TOTAL SCORE	2.935	0.4017
FEV_1_ % predicted	10.350	0.0158
TOTAL EXACERBATIONS	3.768	0.2877
Dyspnea Category	7.121	0.0681
Composite Severity Score	6.453	0.420

Increasing diagnostic delay was associated with progressive increases in pre-diagnostic indicators, including dyspnea, exacerbations, and pharmacological treatment. Patients were grouped into quintiles based on diagnostic delay duration (in days). Kruskal–Wallis H tests showed statistically significant variance across delay groups for most clinical severity indicators, supporting a delay–severity gradient. Higher H values indicate greater between-group variance in severity indicators across delay duration.

**Panel C**. Spearman’s correlation with delay duration (in days).

**Indicator**	**Spearman’s** **ρ**	***p*****-Value**
Frequent PreDx Use	0.47	<0.001
Significant Dyspnea	0.33	<0.001
Frequent Exacerbations	0.42	<0.001
Frequent Treatment Exposure	0.44	<0.001

Moderate positive correlations were observed between delay duration and clinical burden indicators, including dyspnea, exacerbations, and treatment intensity.

**Panel D**. Effect sizes: risk ratios and Cohen’s d for missed vs. not missed.

**Indicator**	**Risk Ratio (RR)**	**Cohen’s d**
Significant Dyspnea	1.54	0.63
Frequent Exacerbations	2.03	0.77

Missed opportunity groups showed moderate-to-large effect sizes for dyspnea and exacerbation burden.

**Panel E.** Chi-Square tests comparing missed vs. not missed diagnostic opportunities across delay bins.

**Indicator**	**Delay Bin Range (Days)**	**χ****^2^**	**df**	***p*****-Value**
Significant Dyspnea	[−42.0, −2.0]	6.21	1	0.013
	[−2.0, 5.2]	5.04	1	0.025
	[24.0, 69.6]	11.83	1	<0.001
	[139.2, 23224.0]	7.15	1	0.007
Frequent Exacerbations	[5.2, 13.2]	4.87	1	0.027
	[69.6, 139.2]	9.65	1	0.002

Statistically significant differences were observed across delay bins, with higher diagnostic delay associated with increased missed opportunities.

**Table A6:** Statistical tests for delay duration analysis—Supplementary Material provided (related to Figure 2 in the main manuscript).

**Indicator**	**Spearman’s** **ρ**	***p*** **-Value**
CAT Score	0.252	0.0011
AVD TOTAL SCORE	0.108	0.1685
FEV_1_ % predicted	−0.201	0.0095
TOTAL EXACERBATIONS	0.106	0.1767
Dyspnea Category	0.106	0.1772
Composite Severity Score	0.063	0.4181

Kruskal–Wallis H tests comparing distributions of severity indicators between binary groups (missed vs. not missed) demonstrated that patients with missed diagnostic opportunities generally exhibited higher clinical severity. Higher scores reflect worse severity, except for FEV_1_ % predicted, where lower values indicate greater impairment.

**Panel A**. Spearman correlation between diagnostic delay duration and clinical severity indicators.

While Spearman’s correlation on continuous delay in days did not show a significant linear relationship for all indicators, a clear dose–response pattern was evident when delay was analyzed across clinically meaningful intervals (as shown in Figure 2 of the main manuscript), suggesting a non-linear or threshold-based effect.

Positive values of Spearman’s ρ indicate that longer diagnostic delay was associated with worse severity profiles, whereas negative values (e.g., for FEV_1_ % predicted) indicate that increasing delay was related to greater impairment. All correlations reflect the relationship between diagnostic delay duration and clinical severity.

**Panel B**. Effect sizes: Cohen’s d and risk ratios for high severity (missed vs. not missed).

**Indicator**	**Cohen’s d**	**Risk Ratio**
CAT Score	−0.626	2.233
AVD_TOTAL_SCORE	−0.190	2.198
FEV1 % predicted	0.265	0.797
TOTAL_EXACERBATIONS	−0.691	16.744
Dyspnea_Category	−0.250	2.326

Negative Cohen’s d indicates higher severity in the “missed opportunities” group. Risk ratios compare the likelihood of above-median severity between missed and non-missed groups.
