# Supplementary material for: Decoding Diagnostic Delay in COPD: An Integrative Analysis of Missed Opportunities, Clinical Risk Profiles, and Targeted Detection Strategies in Primary Care
Source: Diagnostics (Basel). 2025 Aug 30;15(17):2209. doi: 10.3390/diagnostics15172209 (PMC12428083; doi:10.3390/diagnostics15172209)
Supplement: Supplementary file 1 [file diagnostics-15-02209-s001.zip › diagnostics-3815618 Supplementary Figure.pdf]

Supplementary Material

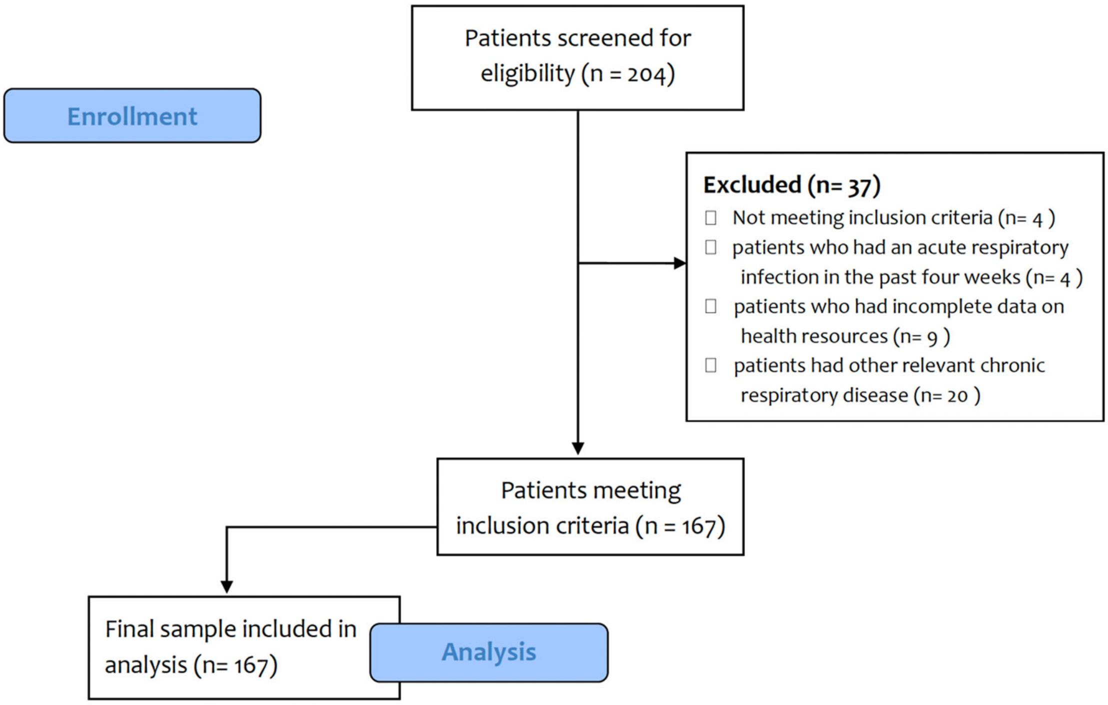

Supplementary Figure S1. Study Flow Diagram (STROBE). Patient inclusion and exclusion criteria and final study population. The figure summarizes the derivation of the analytical cohort from the initial patient pool, including exclusion steps based on diagnostic criteria, comorbid conditions, and data completeness, in accordance with STROBE recommendations for observational studies.
